# The Impacts of Mitoquinone Supplementation on Oxidative Status and Cryo-Survival of Cryopreserved Male Donkey (*Equus asinus*) Semen

**DOI:** 10.3390/vetsci13060510

**Published:** 2026-05-24

**Authors:** Elshymaa A. Abdelnaby, Abdulrhman K. Alhaider, Ibrahim A. Emam

**Affiliations:** Department of Clinical Sciences, College of Veterinary Medicine, King Faisal University, P.O. Box 400, Al-Ahsa 31982, Saudi Arabiaizeater@kfu.edu.sa (I.A.E.)

**Keywords:** male donkey, Mitoquinone, mitochondria, post-thawing, sperm

## Abstract

This current study aimed to determine the impact of Mitoquinone (MitoQ) on the quality of frozen–thawed donkey semen. Ejaculates were collected from six male donkeys (*Equus asinus*), and ejaculates were polled and aliquoted into 12 samples. Samples were diluted with TRIS–egg yolk glycerol extender that reached 200 million sperm/mL. After centrifugation, the pellet was diluted at 1:15 with TRIS–egg yolk glycerol extender and divided into the five main groups containing MitoQ with different concentrations: 0 nmol/mL (control; MitoQ0), 100 nmol/mL (MitoQ1), 150 nmol/mL (MitoQ2), 200 nmol/mL (MitoQ3), and 250 nmol/mL (MitoQ4). After thawing, semen quality was evaluated using CASA kinematic parameters, fluorescence microscopy, and biochemical markers such as alanine and aspartate aminotransferase levels (ALT and AST). Malondialdehyde (MDA) and catalase (CAT) levels were also measured. MitoQ1 with 100 nmol/mL significantly increased total motility (*p* = 0.04), progressive motility (*p* = 0.04), viability (*p* = 0.03), kinetic parameters (*p* = 0.04), linearity (*p* = 0.02), and straightness (*p* = 0.04). A significant decline in MDA (*p* = 0.02), ALT (*p* = 0.03), and AST (*p* = 0.03) levels in MitoQ1 with 100 nmol/mL was found, with an elevation of CAT levels (*p* = 0.02) compared to other concentrations and the control in TRIS–egg yolk glycerol extender. Different concentrations of MitoQ did not affect acrosome and DNA integrity. In conclusion, the addition of MitoQ during cryopreservation has a positive effect on sperm motility, viability, and kinetic parameters, especially at a concentration of 100 nmol/mL when used with a TRIS–egg yolk glycerol extender for frozen–thawed donkey sperm.

## Simple Summary

The actions of different doses of MitoQ on oxidative status and cryo-survival of cryopreserved male donkey semen have been reported. Samples were diluted with TRIS–egg yolk glycerol extender. After centrifugation, the pellet was diluted to 1:15 with TRIS–egg yolk glycerol extender and divided into the five main groups containing MitoQ with different concentrations. MitoQ1 with 100 nmol/mL significantly increased motility, viability, kinetic parameters, linearity, and straightness with a decline in malondialdehyde levels. This study demonstrated that the addition of MitoQ at a concentration of 100 nmol/mL could positively affect motility, viability, and kinetic parameters while reducing malondialdehyde levels in post-thawed donkey sperm.

## 1. Introduction

Donkey breeders use both natural mating and Artificial Insemination (AI) to reach the best reproductive performance, with natural breeding being common in commercial milk production due to lower stress, and AI increasingly used for genetic improvement and overcoming logistical barriers with a critical significance that has led to an increase in livestock production via genetic improvement and breeding [1]. Fertility is usually lower when a female is inseminated with frozen semen instead of fresh semen, primarily due to physical damage from osmotic pressure changes, which lead to the formation of ice crystals, and oxidative stress (OS) caused by excessive production of reactive oxygen species (ROS) that alters sperm quality after thawing [2,3]. In donkeys, the sperm are very sensitive to the accumulation of polyunsaturated fatty acids in the plasma membrane [4,5]. Lower conception rates (0–36%) were presented when frozen–thawed semen was used in donkeys compared to better conception (33–55%) rates in mares [6,7], as this could be related to the inflammatory response that occurs when a jenny is inseminated [8,9,10]. Sperm mitochondria are critical for both physiological processes and cellular metabolism because of ATP production and oxidative phosphorylation [11,12]. Sperm mitochondria could be damaged during freezing, which lowers sperm motility after thawing, as the mitochondrial electron transport chain promotes ROS [13]. In equines, endogenous antioxidants are efficient in combating ROS but remain insufficient to prevent the damage during cryopreservation, leading to decreased sperm viability and fertility rates post-thaw [14].

Recently, researchers have studied the critical role of mitochondria in sperm function maintenance [15]. Some antioxidants were used to protect the mitochondria of sperm from damage during cryopreservation [16,17]. Mitoquinone (MitoQ) is considered an important antioxidant (mitochondria-targeting) and acts as a protective agent for sperm during both freezing and thawing [18,19]. The production of ROS is inhibited by supplementing the semen extender with MitoQ, which can lead to marked increases in the motility percentage, viability percentage, and acrosome integrity of frozen–thawed sperm [20]. Due to its nature as a derivative of Coenzyme Q10, MitoQ is conjugated with a lipophilic triphenyl phosphonium (TPP) cation, accumulates in the mitochondrial matrix by crossing the mitochondrial inner membrane, and then protects sperm [21]. By enhancing mitochondrial function, MitoQ could improve embryo development and fertilization rates, leading to good pregnancy outcomes [22,23]. MitoQ is reduced to ubiquinol (active form), which scavenges hydrogen peroxide by acting on Nrf2/ARE signaling to lower OS [18]. A study in boars reported that the MitoQ supplement (25–200 nmol/L) led to an improvement in the motility percentage, kinetic parameters, acrosome integrity, and DNA integrity in thawed semen [24] with a higher level of catalase and a lower level of malondialdehyde [25]. Positive impacts on semen quality were reported when MitoQ was supplemented in rams [16] and roosters [18]. Despite MitoQ application in stallions [19], its effects on frozen–thawed male donkey semen have not been discussed. Based on the current literature, no data have reported the protective and antioxidant actions of MitoQ on cryopreserved male donkey (*Equus asinus*) semen. Therefore, this current study aimed for the first time to investigate whether MitoQ supplementation with different concentrations could enhance the post-thaw semen quality under cryogenic stress in male donkeys.

## 2. Materials and Methods

This current study was conducted at the Equine stable in the Department of Clinical Sciences, King Faisal University, Al-Hassa, Saudi Arabia during October 2025, with approval from the deanship of the Ethical Committee with an approval number KFU-REC-2025-DEC-ETHICS3903.

### 2.1. Chemicals

Mitoquinone (MitoQ) was purchased from Sigma (MitoQ mesylate; 678.83; AMBH9A6268CA). It was used as an antioxidant in the TRIS–egg yolk glycerol extender at different concentrations to form different groups. All chemicals used were obtained from Sigma-Aldrich (St. Louis, MO, USA).

### 2.2. Preparation of Extender

TRIS–egg yolk glycerol buffer was prepared based on the previous method [26]. The extender formulation contains 3.025 gm of TRIS (hydroxymethyl-aminomethane), 1.7 gm of citric acid (monohydrate), 1.25 gm of fructose, 20 mL of fresh egg yolk, 3–6 mL of glycerol, and antibiotics (Na-benzylpenicillin with 100,000 IU and streptomycin sulfate), then up to 100 mL of double-distilled water. The extender was sterilized and prewarmed at 37 °C in a water bath and mixed gently for 2–3 min before use. While the buffer consisted of 21 mM of HEPES medium combined with skim milk, glucose, disaccharides, and antibacterials, all were dissolved in 73.6 mL of double-distilled water to adjust the pH to 7.2.

On the day of semen collection, 73.6 mL of TRIS basic solution, 6.4 mL of glycerol, and 20 mL of newly prepared egg yolk were combined to create the TRIS–egg yolk glycerol extender, which had a final volume of 100 mL. After removing any remaining egg yolk by centrifuging the extender at 16,000× *g* for 10 min, it was kept at 4 °C.

### 2.3. Experimental Design

This study used six adult healthy male donkeys (*Equus asinus*; n = 6) with a normal semen picture, weighing about 200–400 kg (mean 350 ± 10 kg), aged 7–10 years (mean 8.5 ± 0.5 years), and with body condition scores of about 4 in the Equine stable at the Department of Clinical Sciences, King Faisal University. Males are kept indoors, fed on commercial pelleted ration with hay, and have free access to water. Scrotum, testes, penis, and prepuce were examined [27]. All males were proved to be fertile via previous successful breeding.

### 2.4. Semen Collection and Processing

Before starting the experiment, a mean of 9–11 seminal collections was taken from each male (one collection every two days) to empty the extragonadal sperm reserves. Semen collection was conducted by using a prewarmed (47 °C) and lubricated artificial vagina (AV; Supply Inc., California, MO, USA) with the presence of a jenny in the estrous phase as a mounted animal [28]. After semen sample collection, the ejaculate was immediately transferred to our laboratory and kept at 37 °C for further evaluation. Volume (mL), total motility (TM; %), progressive forward motility (PM; %), and concentrations (10^6^/mL) were examined in freshly collected semen. Sperm quality was examined using a computer-assisted sperm analysis program (CASA system; Songjing Tianlun, Nanjing, China). Only samples showing at least 70 percent progressive motility and 15.2–150.0 million sperm per ml were selected, with no abnormality in color or odor. A total of 30 ejaculates were collected from 6 donkeys over 5 days. Ejaculates were pooled into 12 pools, with each pool including semen from 2.5 ejaculates. Twelve pooled donkey semen samples were diluted with TRIS–egg yolk glycerol extender that reached 200 million sperm/mL, which is atypical for freezing or intensive cryopreservation efforts.

After semen dilution, samples were divided into five groups containing MitoQ with different concentrations: 0 nmol/mL (control; MitoQ_0_), 100 nmol/mL (MitoQ_1_), 150 nmol/mL (MitoQ_2_), 200 nmol/mL (MitoQ_3_), and 250 nmol/mL (MitoQ_4_). Fresh semen samples were centrifuged at 1100 rpm to obtain a sperm pellet and to discard seminal plasma. The obtained pellet was diluted at 1:15 with TRIS–egg yolk glycerol extender and divided into the five main groups.

Semen was placed into 0.5 mL-colored French straws (IMV, Paris, France) containing 250 million total sperm/mL. Then all straws were gradually cooled (from 37 °C to 4 °C at a rate of 1 °C every 3 min). Following the equilibration period, all straws were kept for 15 min in nitrogen vapor for freezing and then stored at −196 °C in a tank of liquid nitrogen. Thawing was done in the vertical thawing unit with standard water baths in a vertical unit (similar to the Mayuresh model) to hold straws vertically (IMV, Made in France) after 24 h from preservation in liquid nitrogen [29,30,31]. Six samples per parameter were measured and evaluated.

### 2.5. Kinematic Sperm Measurement

After thawing of straws at 37 °C, sperm motility and morphology percentages were measured by the CASA program (CASA system; Songjing Tianlun, Nanjing, China). A 20 µm depth Leja chamber was used with 30 frames of images taken at 60 Hz and 5-pixel cell size with 70 minimum contrasts [32,33]. Only samples with 80% TM, 50% PM, and 70% normal morphology were used. CASA settings were as follows: fixed magnification at minus 1.6, 50–60 photometer range, 5-pixel cell size, and 55 intensities. The measured kinematic parameters of progressive sperm were curvilinear velocity (VCL; μm/s), straight-line velocity (VSL; μm/s), path of average velocity (VAP; μm/s), distance of straight line (DSL; μm), distance of curved line (DCL; μm), average path distance (DAP; μm), amplitude of lateral head displacement (ALH; μm), beat cross frequency (BCF; Hz), straightness (STR), and linearity (LIN).

### 2.6. Viability, Acrosome Integrity, and Sperm DNA Integrity Assessment

A modified CFDA (5,6-carboxyfluorescein diacetate) and propidium iodide (PI) staining procedure for equine spermatozoa was used to assess viability. Amounts of 20 µL of CFDA with 10 µL of PI solution were added to 500 µL of the semen sample and 20 µL of formal citrate. The incubation period was 10 min at 37 °C in a dark room to allow enzymatic conversion of CFDA and subsequent PI staining, as described [34]. The assessment was performed via fluorescence microscope (Nikon Eclipse 80i, Niiza, Japan). Positive CFDA and negative PI showed fluorescent green viable intact spermatozoa, while non-viable damaged spermatozoa took on a red-stained head with positive PI. A minimum of 200 spermatozoa were evaluated per sample at 1000× [34].

Estimating acrosome integrity is typically performed using a fluorescence microscope (Nikon Eclipse 80i, Japan) with the peanut agglutinin conjugation staining method (PNA; Sigma-Aldrich, L0770) to detect intact acrosomes. A total of 1 × 10^6^/mL sperm samples were washed three times in phosphate-buffered saline and then centrifuged at 1500 rpm for 10 min, and the formed pellet was re-suspended in PBS. At least 200 spermatozoa across different fields were determined to be intact sperm that appeared with uniform fluorescence, while the damaged sperm appeared with weak or absent fluorescence [35].

Regarding the DNA sperm integrity test, the toluidine blue (TB) staining method was adjusted. It is performed by the detection of both single- and double-stranded DNA breaks with staining of the sperm head metachromatically. The procedures were done according to the previous study in dogs [36], as normal condensed chromatin appeared as bright blue (DNA intact), while the dark or violet sperm head showed fragmentation and instability of chromatin (DNA damaged).

### 2.7. Enzymatic Estimation

To measure malondialdehyde (MDA; µmol/mL) production in equine semen pellets, a determination of lipid peroxidation of sperm membrane was done via Thio barbituric acid reactive substance (TBA). A sperm pellet containing about 108 spermatozoa was combined with TBA for an hour. The sample was kept at 2000 rpm for 10 min with measurement of the supernatant at 535 nm via ultraviolet spectrophotometer [37]. MDA level was determined using the equation 1.56 × 10^5^ mol per cm^3^ as previously determined [38]. Catalase (CAT; U/mg protein) activity was measured in the sperm pellet by determining the degree of H_2_O_2_ degradation using ultraviolet kinetic assay via spectrophotometry as previously measured [39]. Determination was performed at an absorbance of 240 nm, and the measured CAT activity was in U/mg protein.

Alanine aminotransferase levels (ALT; U/L) and aspartate aminotransferase (AST; U/L) were estimated by a commercial kit (Randox Laboratories Ltd., County Antrim, UK). The method for determination was based on ultraviolet kinetics as L-glutamate and oxaloacetate are produced when L-aspartate and α-ketoglutarate react. When malate dehydrogenase and NADH are present, the oxaloacetate is further transformed into NAD. Their activities were determined by ΔA/min × 3376 for AST and ΔA/min × 1746 for ALT, as ΔA/min means absorbance per minute with an absorbance at 340 nm after 60 s and then at 30 s intervals up to 120 s.

### 2.8. Statistical Assessment

All obtained data were presented in tables in Microsoft Excel, and then the IBM SPSS statistical analysis program (IBM SPSS software; version 21) analyzed all data. Data for post-thawing parameters were obtained as mean ± standard error of mean (SEM). The effect of different concentrations of MitoQ was analyzed using ANOVA. Fisher’s Least Significant Difference (LSD) is used as a post hoc test after ANOVA analysis to show the significant difference among examined groups (MitoQ0 [Control; 0 nmol/mL], MitoQ1 [100 nmol/mL], MitoQ2 [150 nmol/mL], MitoQ3 [200 nmol/mL], and MitoQ4 [250 nmol/mL]). The statistical significance was set at a probability value less than 0.05.

## 3. Results

This current investigation studied the effect of Mitoquinone at different concentrations (MitoQ_1_ with 100 nmol/mL, MitoQ_2_ with 150 nmol/mL, MitoQ_3_ with 200 nmol/mL, and MitoQ_4_ with 250 nmol/mL) in TRIS–egg yolk glycerol extender in male donkey (*Equus asinus*) semen compared to normal control (MitoQ_0;_ Control; 0 nmol/mL). Semen picture, sperm kinematics, and enzymatic antioxidant parameters were calculated during cryopreservation.

### 3.1. Semen Picture Assessment

#### 3.1.1. Fresh Semen Sample Evaluation

Ejaculates (n = 30) were collected from six male donkeys (*Equus asinus*), and samples were pooled into only twelve (n = 12). The average fresh semen volume was 86.25 ± 2.55 mL, concentration 412 ± 5.66 10^6^/mL, progressive motility was 75.55 ± 1.55%, and total motility was 84.55 ± 2.55%.

#### 3.1.2. Frozen Semen Sample Evaluation

Frozen semen characteristics were assessed as presented in Table 1. Total motility (TM%), and progressive motility (PM %) were affected by supplementation of Mitoquinone as both were increased significantly (*p* = 0.04 for TM and *p* = 0.01 for PM) at the concentration of MitoQ_1_ with 100 nmol/mL (78.50 ± 2.55% and 29.45 ± 1.05%) compared to other groups and the control, as presented in Table 1. Regarding sperm viability (SV; %; Table 1) that is considered the post-thaw sperm attributes under fluorescent microscopy, there was a significant elevation (*p* = 0.03) in SV% at a concentration of MitoQ_1_ with 100 nmol/mL (45.66 ± 1.55) compared to MitoQ_2_ with 150 nmol/mL, MitoQ_3_ with 200 nmol/mL, and MitoQ_4_ with 250 nmol/mL), and control ((MitoQ_0;_ Control; 0 nmol/mL).

All kinematic parameters in the male donkey (*Equus asinus*) semen were determined via CASA system. Both straight-line velocity (VS; μm/s) and path of average velocity (VAP; μm/s) were increased (*p* = 0.04 for both) at the concentration of MitoQ_1_ with 100 nmol/mL (35.12 ± 2.01 μm/sec and 45.28 ± 2.55 μm/s) compared to other groups and the control as presented in Table 1, while the curvilinear velocity (VCL; μm/s) parameter was similar at all concentrations and the control.

All distance parameters were not significantly affected except the distance of the curved line (DCL;μm); DCL was (*p* = 0.04) increased at the concentration of MitoQ_1_ with 100 nmol/mL (27.65 ± 0.52 μm) compared to MitoQ_2_ with 150 nmol/mL, MitoQ_3_ with 200 nmol/mL, and MitoQ_4_ with 250 nmol/mL), and control ((MitoQ_0;_ Control; 0 nmol/mL). However, the straight line and average path distances (DSL and DAP) were not affected, as presented in Table 1. In addition, both the lateral head displacement amplitude (ALH; μm) and beat cross frequency (BCF; Hz) were not significantly (*p* > 0.05 ) affected. Finally linearity (LIN) and straightness (STR) were increased (*p* = 0.02 for LIN and *p* = 0.03 for STR) at the concentration of MitoQ_1_ with 100 nmol/mL (0.44 ± 0.01 for LIN and 0.78 ± 0.01 for STR) when compared to MitoQ_2_ with 150 nmol/mL, MitoQ_3_ with 200 nmol/mL, and MitoQ_4_ with 250 nmol/mL in TRIS–egg yolk glycerol extender in male donkey (*Equus asinus*) semen and compared to control (MitoQ_0_; Control; 0 nmol/mL). Regarding sperm DNA and acrosome integrity, there was no significant change between different concentrations of MitoQ.

### 3.2. Enzymatic Assessment

As shown in Table 2, a significant decline was determined in malondialdehyde (MDA; nmol/mL; *p* = 0.02), alanine aminotransferase (ALT; U/L; *p* = 0.03), and aspartate aminotransferase (AST; U/L; *p* = 0.03) levels. This decline was observed at MitoQ_1_ with 100 nmol/mL (11.54 ± 1.25 nmol/mL, 2.25 ± 0.04 U/L, and 3.65 ± 0.58 U/L) compared to MitoQ_2_ with 150 nmol/mL (17.25 ± 1.55 nmol/mL, 3.88 ± 0.01 U/L, and 7.01 ± 0.77 U/L), MitoQ_3_ with 200 nmol/mL (17.55 ± 0.74 nmol/mL, 4.01 ± 0.21 U/L, and 6.55 ± 0.54 U/L), MitoQ_4_ with 250 nmol/mL (18.65 ± 2.14 nmol/mL, 3.95 ± 0.01 U/L, and 6.25 ± 0.64 U/L), and control (MitoQ_0_; 0 nmol/mL (22.55 ± 2.36 nmol/mL, 6.55 ± 0.52 U/L, and 8.96 ± 0.47 U/L).

Catalase (CAT; U/mg protein) levels were increased (P = 0.02) at the concentration of MitoQ_1_ with 100 nmol/mL (182.45 ± 11.25 U/mg protein) compared to MitoQ_2_ with 150 nmol/mL, MitoQ_3_ with 200 nmol/mL, and MitoQ^4^ with 250 nmol/mL in TRIS–egg yolk glycerol extender in the semen of male donkeys (*Equus asinus*) and compared to normal control (MitoQ_0_; Control; 0 nmol/mL) as depicted in Table 2.

## 4. Discussion

Equine semen cryopreservation is hindered by temperature alteration, osmotic stress, damage from oxidation, and ice crystal formation. All those obstacles result in lower post-thawing sperm viability (30–50%) compared to other animals [31,40]. Therefore, the addition of external antioxidant substances to the extender could be a highly beneficial strategy to enhance the sperm survival and quality of sperm during storage [41]. Simultaneously, suppression and this oxidative stress are critical for the improvement of post-thaw equine sperm and result in the production of reactive oxygen species (ROS) to cause lipid peroxidation [40]. Mitoquinone (MitoQ) has been widely used due to its antioxidant effects [42]. However, it is unclear whether MitoQ could play a direct role in donkey semen cryopreservation. Therefore, this current study determined the effect of MitoQ addition with different concentrations to the diluent on cryopreserved donkey (*Equus asinus*) semen. The results suggested that MitoQ has a significant positive impact on the improvement of the donkey semen quality and enhancement of its antioxidant ability, with 100 nmol/mL being considered the perfect concentration. Sperm motility, either total or progressive, remains as one of the critical vital metrics for measurement of sperm quality, as only sperm with high quality can transfer into the female reproductive tract, reaching the ampulla and then performing fertilization [43,44]. In addition, studies demonstrated that low pregnancy rates could result from impaired sperm function and malformation [45].

According to this study, adding MitoQ to the extender at a concentration of 100 nmol/L could increase the percentage of viable sperm after freezing and thawing in male donkeys (*Equus asinus*). The mitochondria-targeting antioxidant action, which consists of a lipophilic triphenyl phosphonium (TPP) cation linked to ubiquinone, is the mechanism by which MitoQ may impact motility. This allows good penetration to the phospholipid bilayer and enhances viability and membrane integrity in post-thawed sperm, which leads to complete restoration of mitochondrial function [20,24]. This is in agreement with similar studies that reported the addition of MitoQ, but with a higher concentration (200 nM), could enhance sperm motility [29]. In addition, another study showed an elevation in viability and motility % after the addition of MitoQ in ram semen, and these findings could lead to the enhancement of ewes’ reproductive performance during in vitro fertilization [46]. In addition, the elevation of VAP and VCL in our current study agrees with a study that reported the same elevation and its positive correlation with fertilization rate [46], as high VAP is directly connected with highly motile sperm [19].

This current study reported a significant increase in kinematic parameters measured by CASA, such as distance of curved line (DCL; μm), linearity (LIN), and straightness index (STR) in post-thawed sperm with MitoQ supplement in TRIS–egg yolk glycerol extender with a concentration of 100 nmol/mL compared to other concentrations and the control. Meanwhile, other parameters, such as DAP average path distance (DAP; μm), amplitude of lateral head displacement (ALH; μm), and beat cross frequency (BCF; Hz), did not alter. Similarly, two studies reported supplementation of antioxidants such as proline and hydroxytyrosol in post-thawed sperm enhanced DCL, LIN, and STR, as those parameters are very important in maintaining progressive motility [47,48]. But there are no available data on the action of MitoQ on kinematic parameters. On the contrary, Elkhawagah et al. [19] reported that 25 nM of MitoQ showed a significant increase in ALH (μm) after 30 min of incubation.

Evaluation of enzymatic activity could provide critical information on antioxidant defense mechanisms and membrane integrity [49]. The release of intracellular enzymes reflects the sperm damage in cryopreservation in equines [50]. Total antioxidant capacity (TAC) is used to assess the levels of antioxidants regarding sperm viability and vitality in equines [51]. In addition, the declination of superoxide dismutase (SOD) levels in semen could result in a reduction in animal fertility performance that leads to infertility.

Furthermore, MDA is considered a metabolic product of polyunsaturated fatty acids and is critical in lipid peroxidation [52]. This current study reported that the addition of MitoQ to post-thawed semen extenders with a 100 nmol/mL concentration resulted in a decline in malondialdehyde (MDA; nmol/mL), alanine aminotransferase (ALT; U/L), and aspartate aminotransferase (AST; U/L) levels with an elevation in catalase (CAT; U/mL). This could be explained by MitoQ antioxidants’ ability to increase CAT and SOD levels by a clear reduction in oxidative stress induced by cryopreservation and then the mitigation of the high ROS levels that are present during the thawing process [20,53]. Regarding changes in MDA, ALT, and AST levels, studies showed the protective action of MitoQ, as administration resulted in a decline in ALT and AST by the reduction of liver fat and declination of OS [54]. Similar outcomes were reported when other antioxidants, such as sericin, were used, as it altered ALT activity with a non-significant change in AST with a reduction in MDA levels [55].

It is known that MitoQ plays a direct role in the reduction of ROS generation by activating the signaling pathway of Nrf2/ARE; thus, it reduces the OS levels in the mitochondria and then exerts its antioxidant function in sperm. Similarly, a study showed a positive correlation between SOD, CAT, and MitoQ concentrations and a negative correlation with MDA levels [24]. In addition, it was reported that MitoQ (150 nmol/L) elevates SOD activity in rooster sperm after freezing [18]. In addition to its action on females, the addition of MitoQ in buffalo during in vitro maturation could lead to the improvement of oocytes [42]. The limitation of this study was the lack of other higher concentrations of MitoQ to show its effect on semen quality. Another limitation is a lack of lower dosing of MitoQ concentrations lower than 100 nmol/mL that could affect the semen picture, in addition to the lack of some test for swelling under reduced osmotic pressure (HOS) and abnormality rate.

## 5. Conclusions

In summary, the addition of MitoQ with a glycerol extender made from TRIS and egg yolk is effective in enhancing semen quality (motility, viability, sperm kinetic parameters) with its protective and antioxidant ability. The addition of 100 nmol/L of MitoQ enhances the antioxidant capacity of donkey sperm.

## Figures and Tables

**Table 1:** Traits and kinematic parameters in male donkey (*Equus asinus*) semen subjected to different concentrations of MitoQ supplement in TRIS–egg yolk glycerol extender. Data are obtained as mean ± standard error of mean (SEM).

Variable	MitoQ (nmol/mL) at Different Levels					*p*-Value
	MitoQ_0_ (Control; 0)	MitoQ_1_ (100)	MitoQ_2_ (150)	MitoQ_3_ (200)	MitoQ_4_ (250)	
TM %	71.22 ± 1.55 ^b^	78.50 ± 2.55 ^a^	71.80 ± 1.44 ^b^	72.32 ± 0.22 ^b^	71.22 ± 2.52 ^b^	0.04
PM **%**	19.99 ± 1.05 ^b^	29.45 ± 1.05 ^a^	21.22 ± 1.22 ^b^	22.31 ± 2.55 ^b^	21.55 ± 1.74 ^b^	0.01
SV **%**	40.52 ± 2.32 ^b^	45.66 ± 1.55 ^a^	41.20 ± 1.52 ^b^	40.33 ± 1.25 ^b^	41.65 ± 1.77 ^b^	0.03
VCL μm/s	66.45 ± 1.22	75.68 ± 2.55	69.22 ± 1.25	67.25 ± 1.22	71.25 ± 1.3	0.44
VSL μm/s	20.10 ± 1.05 ^b^	35.12 ± 2.01 ^a^	22.31 ± 0.79 ^b^	21.52 ± 1.44 ^b^	21.00 ± 1.45 ^b^	0.03
VAP μm/s	33.52 ± 1.27 ^b^	45.28 ± 2.55 ^a^	39.52 ± 1.44 ^ab^	31.54 ± 0.69 ^b^	31.22 ± 1.13 ^b^	0.03
DSL μm	11.25 ± 0.33	10.69 ± 0.74	11.32 ± 1.25	10.88 ± 0.88	10.75 ± 0.22	0.55
DCL μm	24.66 ± 0.25 ^b^	27.65 ± 0.52 ^a^	23.55 ± 1.08 ^b^	23.41 ± 0.74 ^b^	22.55 ± 0.52 ^b^	0.04
DAP μm	7.55 ± 0.41	6.95 ± 0.44	7.85 ± 1.07	7.55 ± 0.14	6.55 ± 0.85	0.45
ALH μm	1.33 ± 0.01	1.02 ± 0.01	1.22 ± 0.02	1.14 ± 0.01	1.06 ± 0.21	0.44
BCF Hz	18.65 ± 0.02	19.77 ± 0.44	18.74 ± 1.05	18.99 ± 0.32	19.08 ± 0.55	0.62
LIN (VSL/VCL)	0.31 ± 0.01 ^b^	0.44 ± 0.01 ^a^	0.31 ± 0.01 ^b^	0.32 ± 0.01 ^b^	0.33 ± 0.01 ^b^	0.02
STR (VSL/VAP)	0.61 ± 0.01 ^b^	0.78 ± 0.01 ^a^	0.59 ± 0.01 ^b^	0.64 ± 0.01 ^b^	0.65 ± 0.01 ^b^	0.03
DNA integrity (AO)	91.00 ± 0.02	93.00 ± 1.33	92.22 ± 2.58	93.00 ± 2.74	93.00 ± 3.21	0.35
Ac integrity (FITC-PSA+)	80.55 ± 0.33	81.35 ± 2.33	81.66 ± 5.32	81.65 ± 4.01	82.55 ± 2.22	0.41

TM (%) = total motility, PM(%) = progressive motility, SV (%) = sperm viability, VCL (μm/s) = curvilinear velocity, VSL (μm/s) = straight-line velocity, VAP (μm/s) = path of average velocity, DSL (μm) = distance of straight line, DCL (μm) = distance of curved line, DAP (μm) = average path distance, ALH (μm) = amplitude of lateral head displacement, BCF (Hz) = beat cross frequency, STR = straightness, and LIN= linearity, Ac = sperm acrosome integrity. Means with different superscripts indicate a significant difference between rows at *p* less than 0.05.

**Table 2:** Enzymatic activity parameters in male donkey (*Equus asinus*) semen at different concentrations of MitoQ supplement in TRIS–egg yolk glycerol extender. Data are obtained as mean ± standard error of mean (SEM).

Variable	MitoQ (nmol/mL) at Different Levels					*p*-Value
	MitoQ_0_ (Control; 0%)	MitoQ_1_ (100)	MitoQ_2_ (150)	MitoQ_3_ (200)	MitoQ_4_ (250)	
MDA (µmol/mL)	22.55 ± 2.36 ^b^	11.54 ± 1.25 ^a^	17.25 ± 1.55 ^ab^	17.55 ± 0.74 ^ab^	18.65 ± 2.14 ^ab^	0.02
CAT (U/mg protein)	131.21 ± 15.25 ^b^	182.45 ± 11.25 ^a^	156.41 ± 6.85 ^ab^	155.41 ± 9.12 ^ab^	145.44 ± 9.66 ^ab^	0.02
ALT (U/L)	6.55 ± 0.52 ^b^	2.25 ± 0.04 ^a^	3.88 ± 0.01 ^ab^	4.01 ± 0.21 ^ab^	3.95 ± 0.01 ^ab^	0.03
AST (U/L)	8.96 ± 0.47 ^b^	3.65 ± 0.58 ^a^	7.01 ± 0.77 ^ab^	6.55 ± 0.54 ^ab^	6.25 ± 0.64 ^ab^	0.03

MDA (µmol/mL) = malondialdehyde, CAT (U/mg protein) = catalase, ALT (U/L) = alanine aminotransferase, and AST (U/L) = aspartate aminotransferase. Means with different superscripts indicate a significant difference between rows at *p* less than 0.05.

## Data Availability

The data presented in this study are available on request from the corresponding author. The data are not publicly available due to privacy.
